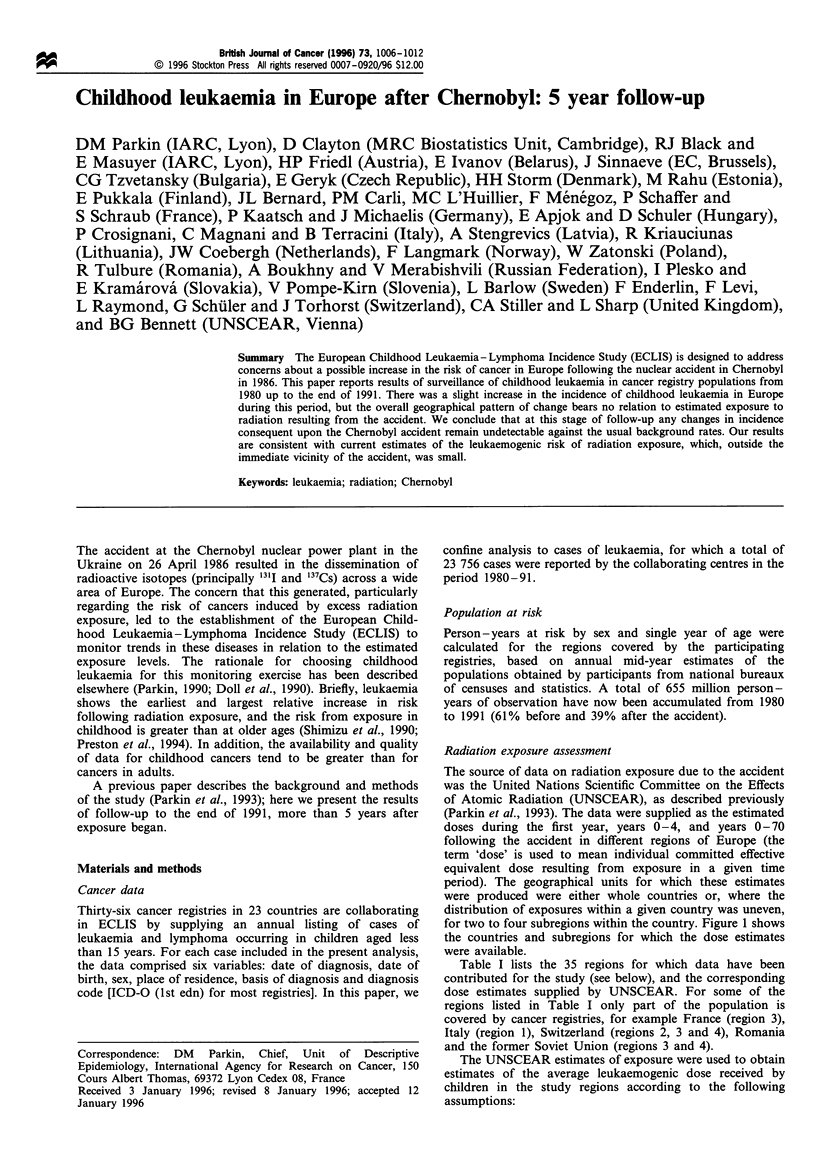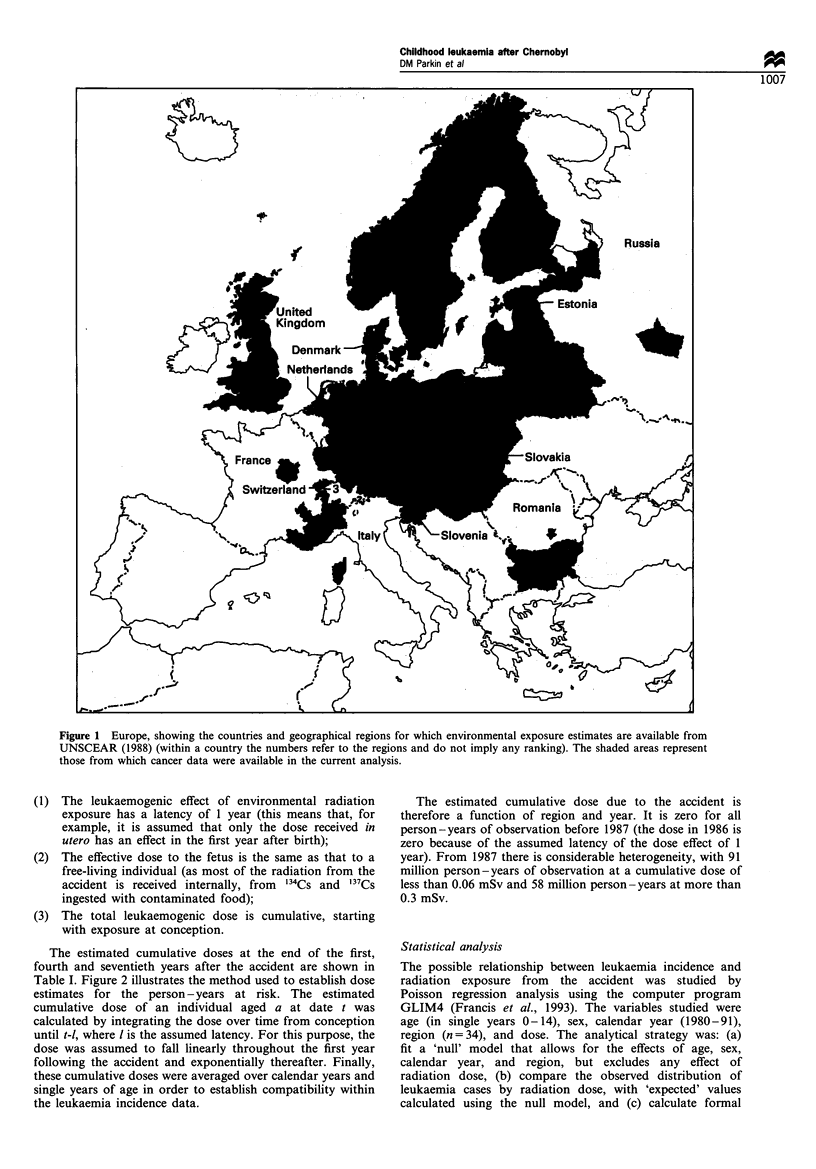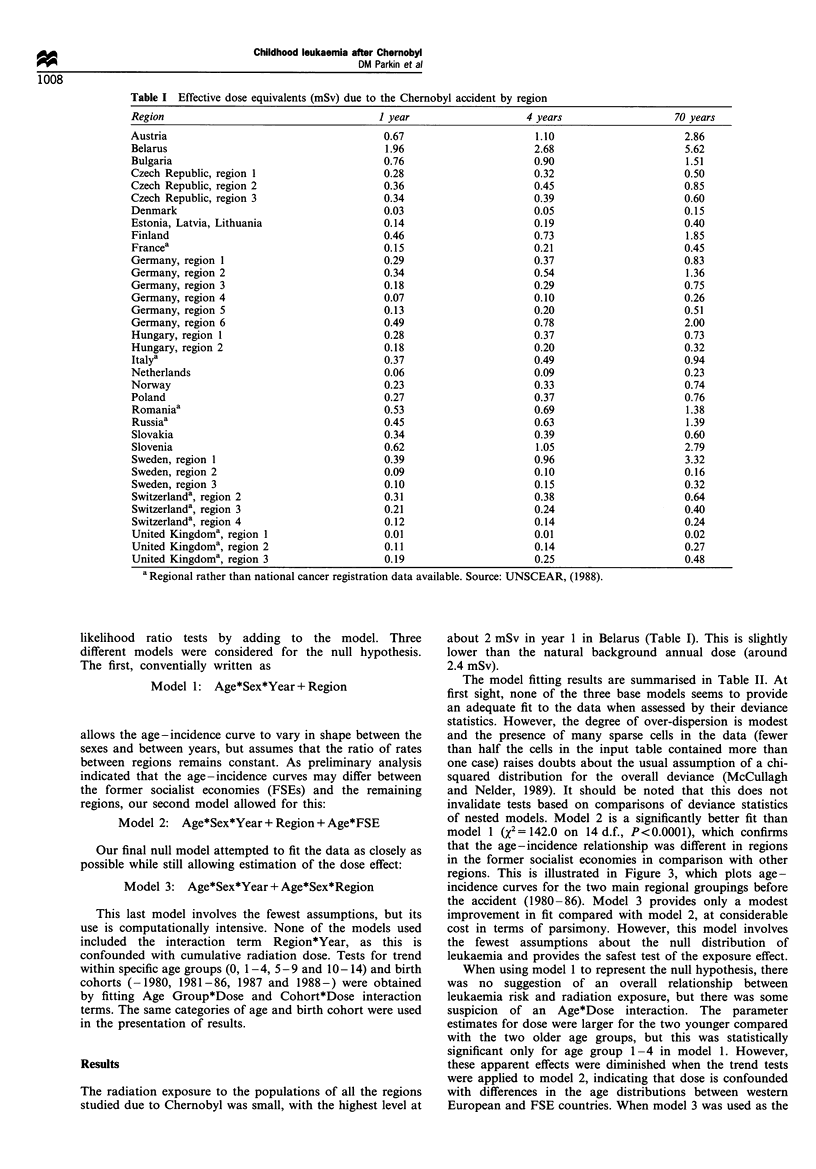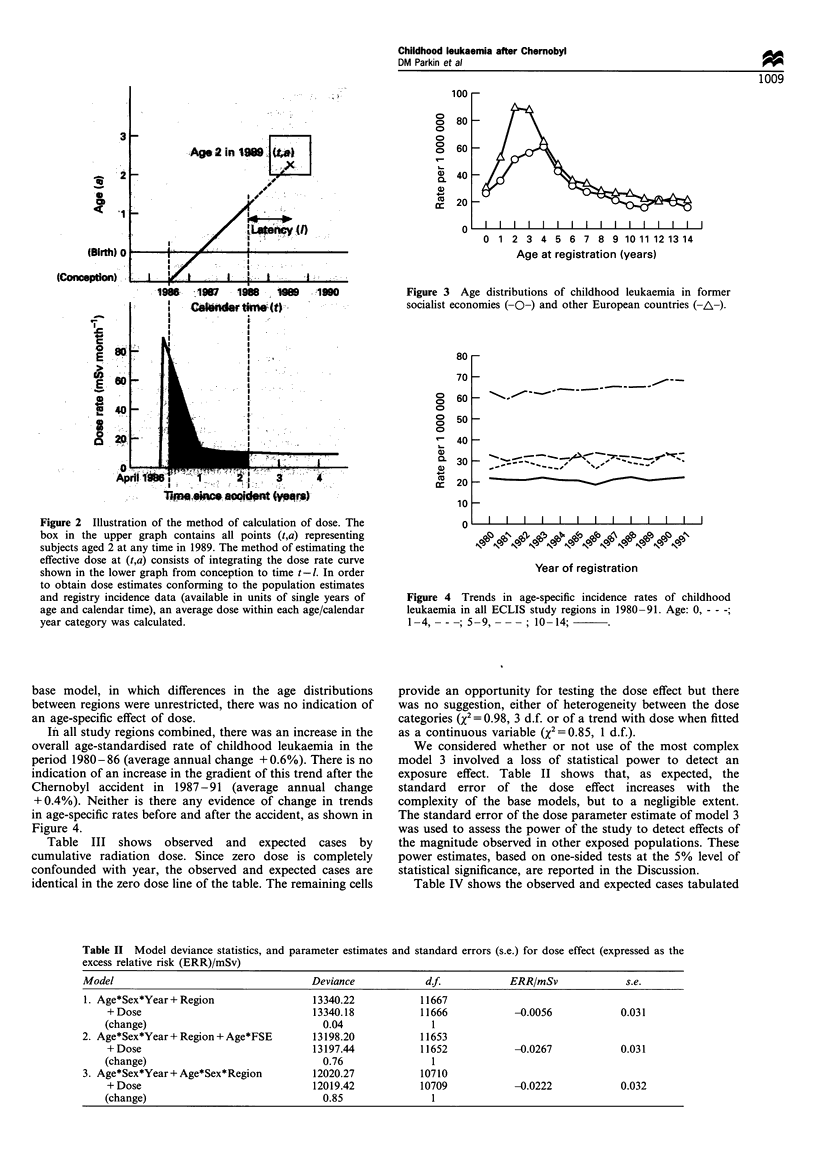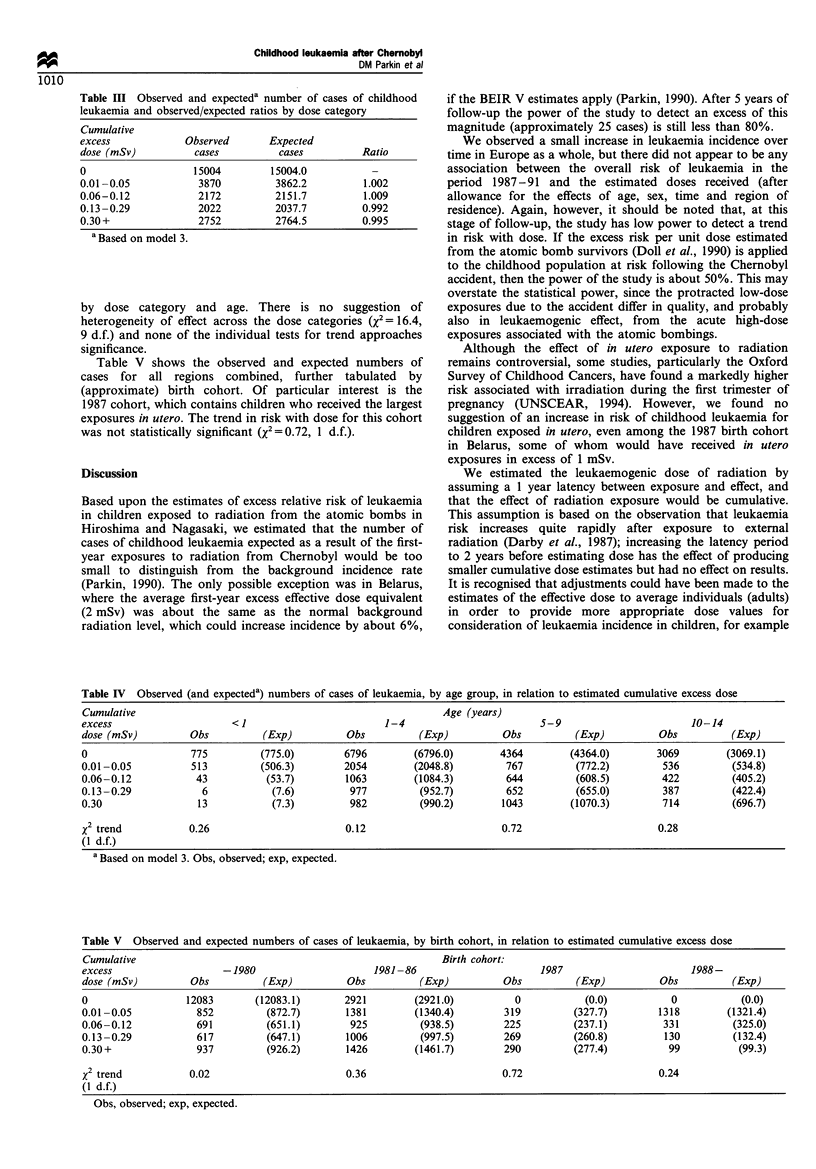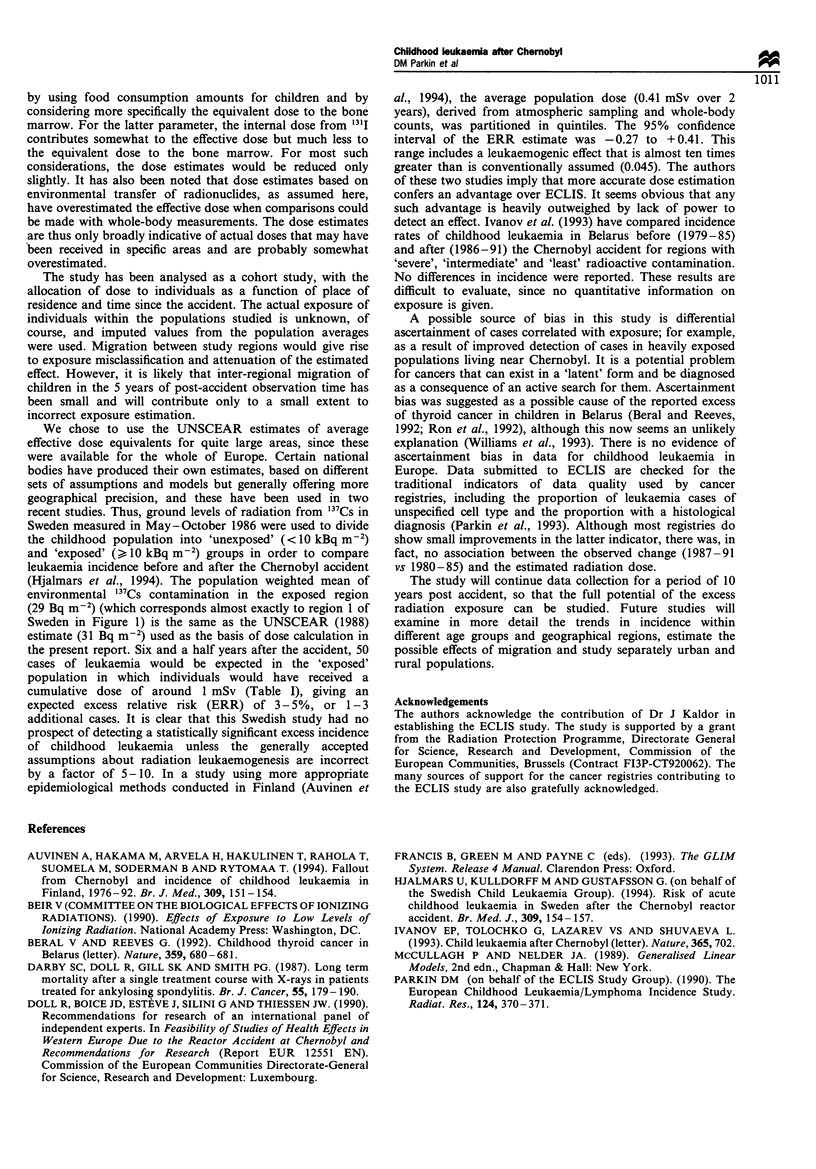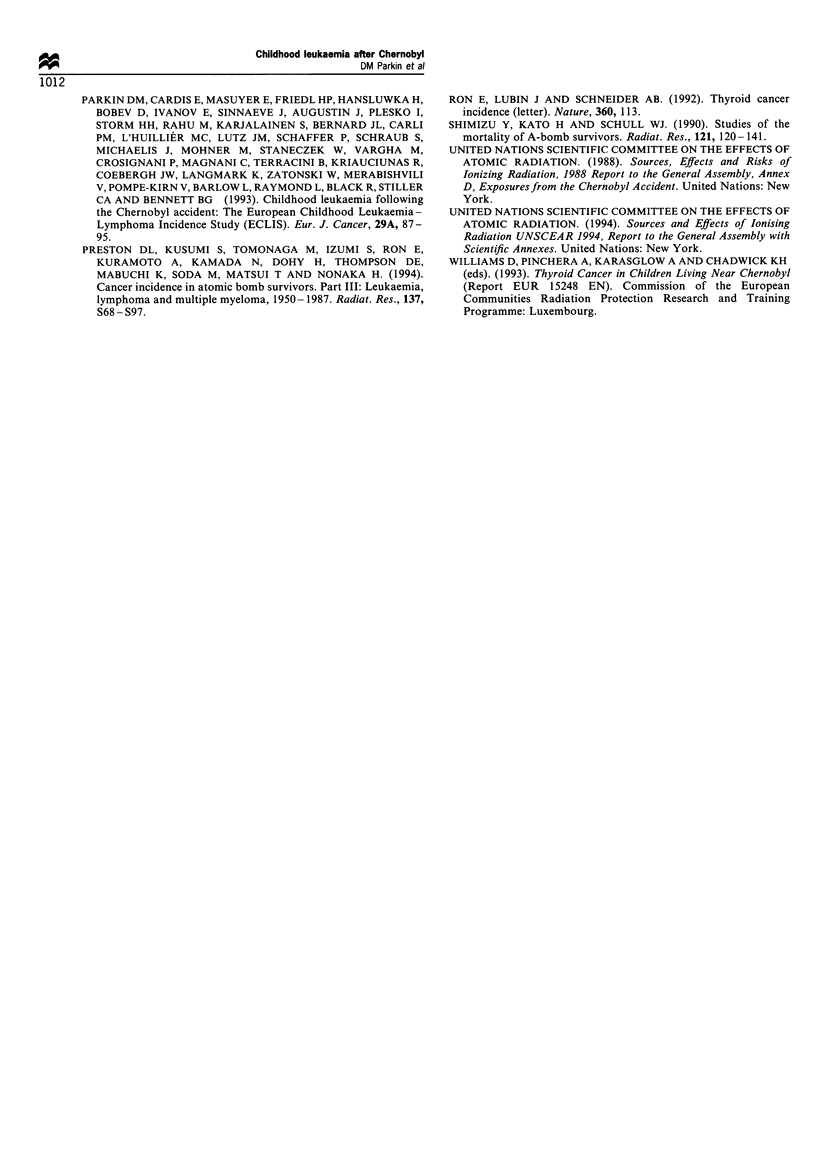# Childhood leukaemia in Europe after Chernobyl: 5 year follow-up.

**DOI:** 10.1038/bjc.1996.197

**Published:** 1996-04

**Authors:** D. M. Parkin, D. Clayton, R. J. Black, E. Masuyer, H. P. Friedl, E. Ivanov, J. Sinnaeve, C. G. Tzvetansky, E. Geryk, H. H. Storm, M. Rahu, E. Pukkala, J. L. Bernard, P. M. Carli, M. C. L'Huilluier, F. Ménégoz, P. Schaffer, S. Schraub, P. Kaatsch, J. Michaelis, E. Apjok, D. Schuler, P. Crosignani, C. Magnani, B. G. Bennett

**Affiliations:** Unit of Descriptive Epidemiology, International Agency for Research on Cancer, Lyon, France.

## Abstract

The European Childhood Leukaemia - Lymphoma Incidence Study (ECLIS) is designed to address concerns about a possible increase in the risk of cancer in Europe following the nuclear accident in Chernobyle in 1986. This paper reports results of surveillance of childhood leukaemia in cancer registry populations from 1980 up to the end of 1991. There was a slight increase in the incidence of childhood leukaemia in Europe during this period, but the overall geographical pattern of change bears no relation to estimated exposure to radiation resulting from the accident. We conclude that at this stage of follow-up any changes in incidence consequent upon the Chernobyl accident remain undetectable against the usual background rates. Our results are consistent with current estimates of the leukaemogenic risk of radiation exposure, which, outside the immediate vicinity of the accident, was small.


British Journal of Cancer (1996) 73, 1006–1012
© 1996 Stockton Press All rights reserved 0007–0920/96 $12.00

### Childhood leukaemia in Europe after Chernobyl: 5 year follow-up

DM Parkin (IARC, Lyon), D Clayton (MRC Biostatistics Unit, Cambridge), RJ Black and E Masuyer (IARC, Lyon), HP Friedl (Austria), E Ivanov (Belarus), J Sinnaeve (EC, Brussels), CG Tzvetansky (Bulgaria), E Geryk (Czech Republic), HH Storm (Denmark), M Rahu (Estonia), E Pukkala (Finland), JL Bernard, PM Carli, MC L'Huillier, F Ménégoz, P Schaffer and S Schraub (France), P Kaatsch and J Michaelis (Germany), E Apjok and D Schuler (Hungary), P Crosignani, C Magnani and B Terracini (Italy), A Stengrevics (Latvia), R Kriauciunas (Lithuania), JW Coebergh (Netherlands), F Langmark (Norway), W Zatonski (Poland), R Tulbure (Romania), A Boukhny and V Merabishvili (Russian Federation), I Plesko and E Kramárová (Slovakia), V Pompe-Kirn (Slovenia), L Barlow (Sweden) F Enderlin, F Levi, L Raymond, G Schüler and J Torhorst (Switzerland), CA Stiller and L Sharp (United Kingdom), and BG Bennett (UNSCEAR, Vienna)

**Summary** The European Childhood Leukaemia–Lymphoma Incidence Study (ECLIS) is designed to address concerns about a possible increase in the risk of cancer in Europe following the nuclear accident in Chernobyl in 1986. This paper reports results of surveillance of childhood leukaemia in cancer registry populations from 1980 up to the end of 1991. There was a slight increase in the incidence of childhood leukaemia in Europe during this period, but the overall geographical pattern of change bears no relation to estimated exposure to radiation resulting from the accident. We conclude that at this stage of follow-up any changes in incidence consequent upon the Chernobyl accident remain undetectable against the usual background rates. Our results are consistent with current estimates of the leukaemogenic risk of radiation exposure, which, outside the immediate vicinity of the accident, was small.

**Keywords:** leukaemia; radiation; Chernobyl

The accident at the Chernobyl nuclear power plant in the Ukraine on 26 April 1986 resulted in the dissemination of radioactive isotopes (principally $^{131}$I and $^{137}$Cs) across a wide area of Europe. The concern that this generated, particularly regarding the risk of cancers induced by excess radiation exposure, led to the establishment of the European Childhood Leukaemia–Lymphoma Incidence Study (ECLIS) to monitor trends in these diseases in relation to the estimated exposure levels. The rationale for choosing childhood leukaemia for this monitoring exercise has been described elsewhere (Parkin, 1990; Doll *et al.*, 1990). Briefly, leukaemia shows the earliest and largest relative increase in risk following radiation exposure, and the risk from exposure in childhood is greater than at older ages (Shimizu *et al.*, 1990; Preston *et al.*, 1994). In addition, the availability and quality of data for childhood cancers tend to be greater than for cancers in adults.

A previous paper describes the background and methods of the study (Parkin *et al.*, 1993); here we present the results of follow-up to the end of 1991, more than 5 years after exposure began.

### Materials and methods

### *Cancer data*

Thirty-six cancer registries in 23 countries are collaborating in ECLIS by supplying an annual listing of cases of leukaemia and lymphoma occurring in children aged less than 15 years. For each case included in the present analysis, the data comprised six variables: date of diagnosis, date of birth, sex, place of residence, basis of diagnosis and diagnosis code [ICD-O (1st edn) for most registries]. In this paper, we confine analysis to cases of leukaemia, for which a total of 23 756 cases were reported by the collaborating centres in the period 1980–91.

### *Population at risk*

Person–years at risk by sex and single year of age were calculated for the regions covered by the participating registries, based on annual mid-year estimates of the populations obtained by participants from national bureaux of censuses and statistics. A total of 655 million person–years of observation have now been accumulated from 1980 to 1991 (61% before and 39% after the accident).

### *Radiation exposure assessment*

The source of data on radiation exposure due to the accident was the United Nations Scientific Committee on the Effects of Atomic Radiation (UNSCEAR), as described previously (Parkin *et al.*, 1993). The data were supplied as the estimated doses during the first year, years 0–4, and years 0–70 following the accident in different regions of Europe (the term 'dose' is used to mean individual committed effective equivalent dose resulting from exposure in a given time period). The geographical units for which these estimates were produced were either whole countries or, where the distribution of exposures within a given country was uneven, for two to four subregions within the country. Figure 1 shows the countries and subregions for which the dose estimates were available.

Table I lists the 35 regions for which data have been contributed for the study (see below), and the corresponding dose estimates supplied by UNSCEAR. For some of the regions listed in Table I only part of the population is covered by cancer registries, for example France (region 3), Italy (region 1), Switzerland (regions 2, 3 and 4), Romania and the former Soviet Union (regions 3 and 4).

The UNSCEAR estimates of exposure were used to obtain estimates of the average leukaemogenic dose received by children in the study regions according to the following assumptions:

Correspondence: DM Parkin, Chief, Unit of Descriptive Epidemiology, International Agency for Research on Cancer, 150 Cours Albert Thomas, 69372 Lyon Cedex 08, France
Received 3 January 1996; revised 8 January 1996; accepted 12 January 1996

**Figure 1** Europe, showing the countries and geographical regions for which environmental exposure estimates are available from UNSCEAR (1988) (within a country the numbers refer to the regions and do not imply any ranking). The shaded areas represent those from which cancer data were available in the current analysis.

(1) The leukaemogenic effect of environmental radiation exposure has a latency of 1 year (this means that, for example, it is assumed that only the dose received *in utero* has an effect in the first year after birth);

(2) The effective dose to the fetus is the same as that to a free-living individual (as most of the radiation from the accident is received internally, from $^{134}Cs$ and $^{137}Cs$ ingested with contaminated food);

(3) The total leukaemogenic dose is cumulative, starting with exposure at conception.

The estimated cumulative doses at the end of the first, fourth and seventieth years after the accident are shown in Table I. Figure 2 illustrates the method used to establish dose estimates for the person–years at risk. The estimated cumulative dose of an individual aged $a$ at date $t$ was calculated by integrating the dose over time from conception until $t$-$l$, where $l$ is the assumed latency. For this purpose, the dose was assumed to fall linearly throughout the first year following the accident and exponentially thereafter. Finally, these cumulative doses were averaged over calendar years and single years of age in order to establish compatibility within the leukaemia incidence data.

The estimated cumulative dose due to the accident is therefore a function of region and year. It is zero for all person–years of observation before 1987 (the dose in 1986 is zero because of the assumed latency of the dose effect of 1 year). From 1987 there is considerable heterogeneity, with 91 million person–years of observation at a cumulative dose of less than 0.06 mSv and 58 million person–years at more than 0.3 mSv.

### *Statistical analysis*

The possible relationship between leukaemia incidence and radiation exposure from the accident was studied by Poisson regression analysis using the computer program GLIM4 (Francis *et al.*, 1993). The variables studied were age (in single years 0–14), sex, calendar year (1980–91), region ($n = 34$), and dose. The analytical strategy was: (a) fit a 'null' model that allows for the effects of age, sex, calendar year, and region, but excludes any effect of radiation dose, (b) compare the observed distribution of leukaemia cases by radiation dose, with 'expected' values calculated using the null model, and (c) calculate formal

**Table I** Effective dose equivalents (mSv) due to the Chernobyl accident by region

| *Region* | *1 year* | *4 years* | *70 years* |
|---|---|---|---|
| Austria | 0.67 | 1.10 | 2.86 |
| Belarus | 1.96 | 2.68 | 5.62 |
| Bulgaria | 0.76 | 0.90 | 1.51 |
| Czech Republic, region 1 | 0.28 | 0.32 | 0.50 |
| Czech Republic, region 2 | 0.36 | 0.45 | 0.85 |
| Czech Republic, region 3 | 0.34 | 0.39 | 0.60 |
| Denmark | 0.03 | 0.05 | 0.15 |
| Estonia, Latvia, Lithuania | 0.14 | 0.19 | 0.40 |
| Finland | 0.46 | 0.73 | 1.85 |
| France[a] | 0.15 | 0.21 | 0.45 |
| Germany, region 1 | 0.29 | 0.37 | 0.83 |
| Germany, region 2 | 0.34 | 0.54 | 1.36 |
| Germany, region 3 | 0.18 | 0.29 | 0.75 |
| Germany, region 4 | 0.07 | 0.10 | 0.26 |
| Germany, region 5 | 0.13 | 0.20 | 0.51 |
| Germany, region 6 | 0.49 | 0.78 | 2.00 |
| Hungary, region 1 | 0.28 | 0.37 | 0.73 |
| Hungary, region 2 | 0.18 | 0.20 | 0.32 |
| Italy[a] | 0.37 | 0.49 | 0.94 |
| Netherlands | 0.06 | 0.09 | 0.23 |
| Norway | 0.23 | 0.33 | 0.74 |
| Poland | 0.27 | 0.37 | 0.76 |
| Romania[a] | 0.53 | 0.69 | 1.38 |
| Russia[a] | 0.45 | 0.63 | 1.39 |
| Slovakia | 0.34 | 0.39 | 0.60 |
| Slovenia | 0.62 | 1.05 | 2.79 |
| Sweden, region 1 | 0.39 | 0.96 | 3.32 |
| Sweden, region 2 | 0.09 | 0.10 | 0.16 |
| Sweden, region 3 | 0.10 | 0.15 | 0.32 |
| Switzerland[a], region 2 | 0.31 | 0.38 | 0.64 |
| Switzerland[a], region 3 | 0.21 | 0.24 | 0.40 |
| Switzerland[a], region 4 | 0.12 | 0.14 | 0.24 |
| United Kingdom[a], region 1 | 0.01 | 0.01 | 0.02 |
| United Kingdom[a], region 2 | 0.11 | 0.14 | 0.27 |
| United Kingdom[a], region 3 | 0.19 | 0.25 | 0.48 |

[a] Regional rather than national cancer registration data available. Source: UNSCEAR, (1988).

likelihood ratio tests by adding to the model. Three different models were considered for the null hypothesis. The first, conventially written as

Model 1: Age*Sex*Year + Region allows the age–incidence curve to vary in shape between the sexes and between years, but assumes that the ratio of rates between regions remains constant. As preliminary analysis indicated that the age–incidence curves may differ between the former socialist economies (FSEs) and the remaining regions, our second model allowed for this:

Model 2: Age*Sex*Year + Region + Age*FSE

Our final null model attempted to fit the data as closely as possible while still allowing estimation of the dose effect:

Model 3: Age*Sex*Year + Age*Sex*Region

This last model involves the fewest assumptions, but its use is computationally intensive. None of the models used included the interaction term Region*Year, as this is confounded with cumulative radiation dose. Tests for trend within specific age groups (0, 1–4, 5–9 and 10–14) and birth cohorts (–1980, 1981–86, 1987 and 1988–) were obtained by fitting Age Group*Dose and Cohort*Dose interaction terms. The same categories of age and birth cohort were used in the presentation of results.

### Results

The radiation exposure to the populations of all the regions studied due to Chernobyl was small, with the highest level at about 2 mSv in year 1 in Belarus (Table I). This is slightly lower than the natural background annual dose (around 2.4 mSv).

The model fitting results are summarised in Table II. At first sight, none of the three base models seems to provide an adequate fit to the data when assessed by their deviance statistics. However, the degree of over-dispersion is modest and the presence of many sparse cells in the data (fewer than half the cells in the input table contained more than one case) raises doubts about the usual assumption of a chi-squared distribution for the overall deviance (McCullagh and Nelder, 1989). It should be noted that this does not invalidate tests based on comparisons of deviance statistics of nested models. Model 2 is a significantly better fit than model 1 ($\chi^2 = 142.0$ on 14 d.f., $P < 0.0001$), which confirms that the age–incidence relationship was different in regions in the former socialist economies in comparison with other regions. This is illustrated in Figure 3, which plots age–incidence curves for the two main regional groupings before the accident (1980–86). Model 3 provides only a modest improvement in fit compared with model 2, at considerable cost in terms of parsimony. However, this model involves the fewest assumptions about the null distribution of leukaemia and provides the safest test of the exposure effect.

When using model 1 to represent the null hypothesis, there was no suggestion of an overall relationship between leukaemia risk and radiation exposure, but there was some suspicion of an Age*Dose interaction. The parameter estimates for dose were larger for the two younger compared with the two older age groups, but this was statistically significant only for age group 1–4 in model 1. However, these apparent effects were diminished when the trend tests were applied to model 2, indicating that dose is confounded with differences in the age distributions between western European and FSE countries. When model 3 was used as the

**Figure 2** Illustration of the method of calculation of dose. The box in the upper graph contains all points $(t,a)$ representing subjects aged 2 at any time in 1989. The method of estimating the effective dose at $(t,a)$ consists of integrating the dose rate curve shown in the lower graph from conception to time $t-l$. In order to obtain dose estimates conforming to the population estimates and registry incidence data (available in units of single years of age and calendar time), an average dose within each age/calendar year category was calculated.

**Figure 3** Age distributions of childhood leukaemia in former socialist economies (–○–) and other European countries (–△–).

**Figure 4** Trends in age-specific incidence rates of childhood leukaemia in all ECLIS study regions in 1980–91. Age: 0, - - -; 1–4, – – –; 5–9, — — — ; 10–14; ———.

base model, in which differences in the age distributions between regions were unrestricted, there was no indication of an age-specific effect of dose.

In all study regions combined, there was an increase in the overall age-standardised rate of childhood leukaemia in the period 1980–86 (average annual change +0.6%). There is no indication of an increase in the gradient of this trend after the Chernobyl accident in 1987–91 (average annual change +0.4%). Neither is there any evidence of change in trends in age-specific rates before and after the accident, as shown in Figure 4.

Table III shows observed and expected cases by cumulative radiation dose. Since zero dose is completely confounded with year, the observed and expected cases are identical in the zero dose line of the table. The remaining cells provide an opportunity for testing the dose effect but there was no suggestion, either of heterogeneity between the dose categories ($\chi^2=0.98$, 3 d.f. or of a trend with dose when fitted as a continuous variable ($\chi^2=0.85$, 1 d.f.).

We considered whether or not use of the most complex model 3 involved a loss of statistical power to detect an exposure effect. Table II shows that, as expected, the standard error of the dose effect increases with the complexity of the base models, but to a negligible extent. The standard error of the dose parameter estimate of model 3 was used to assess the power of the study to detect effects of the magnitude observed in other exposed populations. These power estimates, based on one-sided tests at the 5% level of statistical significance, are reported in the Discussion.

Table IV shows the observed and expected cases tabulated

**Table II** Model deviance statistics, and parameter estimates and standard errors (s.e.) for dose effect (expressed as the excess relative risk (ERR)/mSv)

| *Model* | *Deviance* | *d.f.* | *ERR/mSv* | *s.e.* |
|---|---|---|---|---|
| 1. Age*Sex*Year + Region | 13340.22 | 11667 | | |
| + Dose | 13340.18 | 11666 | –0.0056 | 0.031 |
| (change) | 0.04 | 1 | | |
| 2. Age*Sex*Year + Region + Age*FSE | 13198.20 | 11653 | | |
| + Dose | 13197.44 | 11652 | –0.0267 | 0.031 |
| (change) | 0.76 | 1 | | |
| 3. Age*Sex*Year + Age*Sex*Region | 12020.27 | 10710 | | |
| + Dose | 12019.42 | 10709 | –0.0222 | 0.032 |
| (change) | 0.85 | 1 | | |

**Table III** Observed and expected[a] number of cases of childhood leukaemia and observed/expected ratios by dose category

| *Cumulative excess dose (mSv)* | *Observed cases* | *Expected cases* | *Ratio* |
|---|---|---|---|
| 0 | 15004 | 15004.0 | – |
| 0.01–0.05 | 3870 | 3862.2 | 1.002 |
| 0.06–0.12 | 2172 | 2151.7 | 1.009 |
| 0.13–0.29 | 2022 | 2037.7 | 0.992 |
| 0.30+ | 2752 | 2764.5 | 0.995 |

[a] Based on model 3.

by dose category and age. There is no suggestion of heterogeneity of effect across the dose categories ($\chi^2=16.4$, 9 d.f.) and none of the individual tests for trend approaches significance.

Table V shows the observed and expected numbers of cases for all regions combined, further tabulated by (approximate) birth cohort. Of particular interest is the 1987 cohort, which contains children who received the largest exposures *in utero*. The trend in risk with dose for this cohort was not statistically significant ($\chi^2=0.72$, 1 d.f.).

### Discussion

Based upon the estimates of excess relative risk of leukaemia in children exposed to radiation from the atomic bombs in Hiroshima and Nagasaki, we estimated that the number of cases of childhood leukaemia expected as a result of the first-year exposures to radiation from Chernobyl would be too small to distinguish from the background incidence rate (Parkin, 1990). The only possible exception was in Belarus, where the average first-year excess effective dose equivalent (2 mSv) was about the same as the normal background radiation level, which could increase incidence by about 6%, if the BEIR V estimates apply (Parkin, 1990). After 5 years of follow-up the power of the study to detect an excess of this magnitude (approximately 25 cases) is still less than 80%.

We observed a small increase in leukaemia incidence over time in Europe as a whole, but there did not appear to be any association between the overall risk of leukaemia in the period 1987–91 and the estimated doses received (after allowance for the effects of age, sex, time and region of residence). Again, however, it should be noted that, at this stage of follow-up, the study has low power to detect a trend in risk with dose. If the excess risk per unit dose estimated from the atomic bomb survivors (Doll *et al.*, 1990) is applied to the childhood population at risk following the Chernobyl accident, then the power of the study is about 50%. This may overstate the statistical power, since the protracted low-dose exposures due to the accident differ in quality, and probably also in leukaemogenic effect, from the acute high-dose exposures associated with the atomic bombings.

Although the effect of *in utero* exposure to radiation remains controversial, some studies, particularly the Oxford Survey of Childhood Cancers, have found a markedly higher risk associated with irradiation during the first trimester of pregnancy (UNSCEAR, 1994). However, we found no suggestion of an increase in risk of childhood leukaemia for children exposed *in utero*, even among the 1987 birth cohort in Belarus, some of whom would have received *in utero* exposures in excess of 1 mSv.

We estimated the leukaemogenic dose of radiation by assuming a 1 year latency between exposure and effect, and that the effect of radiation exposure would be cumulative. This assumption is based on the observation that leukaemia risk increases quite rapidly after exposure to external radiation (Darby *et al.*, 1987); increasing the latency period to 2 years before estimating dose has the effect of producing smaller cumulative dose estimates but had no effect on results. It is recognised that adjustments could have been made to the estimates of the effective dose to average individuals (adults) in order to provide more appropriate dose values for consideration of leukaemia incidence in children, for example

**Table IV** Observed (and expected[a]) numbers of cases of leukaemia, by age group, in relation to estimated cumulative excess dose

| *Cumulative excess dose (mSv)* | *Age (years)* <1 Obs | <1 (Exp) | 1–4 Obs | 1–4 (Exp) | 5–9 Obs | 5–9 (Exp) | 10–14 Obs | 10–14 (Exp) |
|---|---|---|---|---|---|---|---|---|
| 0 | 775 | (775.0) | 6796 | (6796.0) | 4364 | (4364.0) | 3069 | (3069.1) |
| 0.01–0.05 | 513 | (506.3) | 2054 | (2048.8) | 767 | (772.2) | 536 | (534.8) |
| 0.06–0.12 | 43 | (53.7) | 1063 | (1084.3) | 644 | (608.5) | 422 | (405.2) |
| 0.13–0.29 | 6 | (7.6) | 977 | (952.7) | 652 | (655.0) | 387 | (422.4) |
| 0.30 | 13 | (7.3) | 982 | (990.2) | 1043 | (1070.3) | 714 | (696.7) |
| $\chi^2$ trend (1 d.f.) | 0.26 | | 0.12 | | 0.72 | | 0.28 | |

[a] Based on model 3. Obs, observed; exp, expected.

**Table V** Observed and expected numbers of cases of leukaemia, by birth cohort, in relation to estimated cumulative excess dose

| *Cumulative excess dose (mSv)* | *Birth cohort:* –1980 Obs | –1980 (Exp) | 1981–86 Obs | 1981–86 (Exp) | 1987 Obs | 1987 (Exp) | 1988– Obs | 1988– (Exp) |
|---|---|---|---|---|---|---|---|---|
| 0 | 12083 | (12083.1) | 2921 | (2921.0) | 0 | (0.0) | 0 | (0.0) |
| 0.01–0.05 | 852 | (872.7) | 1381 | (1340.4) | 319 | (327.7) | 1318 | (1321.4) |
| 0.06–0.12 | 691 | (651.1) | 925 | (938.5) | 225 | (237.1) | 331 | (325.0) |
| 0.13–0.29 | 617 | (647.1) | 1006 | (997.5) | 269 | (260.8) | 130 | (132.4) |
| 0.30+ | 937 | (926.2) | 1426 | (1461.7) | 290 | (277.4) | 99 | (99.3) |
| $\chi^2$ trend (1 d.f.) | 0.02 | | 0.36 | | 0.72 | | 0.24 | |

Obs, observed; exp, expected.

by using food consumption amounts for children and by considering more specifically the equivalent dose to the bone marrow. For the latter parameter, the internal dose from $^{131}I$ contributes somewhat to the effective dose but much less to the equivalent dose to the bone marrow. For most such considerations, the dose estimates would be reduced only slightly. It has also been noted that dose estimates based on environmental transfer of radionuclides, as assumed here, have overestimated the effective dose when comparisons could be made with whole-body measurements. The dose estimates are thus only broadly indicative of actual doses that may have been received in specific areas and are probably somewhat overestimated.

The study has been analysed as a cohort study, with the allocation of dose to individuals as a function of place of residence and time since the accident. The actual exposure of individuals within the populations studied is unknown, of course, and imputed values from the population averages were used. Migration between study regions would give rise to exposure misclassification and attenuation of the estimated effect. However, it is likely that inter-regional migration of children in the 5 years of post-accident observation time has been small and will contribute only to a small extent to incorrect exposure estimation.

We chose to use the UNSCEAR estimates of average effective dose equivalents for quite large areas, since these were available for the whole of Europe. Certain national bodies have produced their own estimates, based on different sets of assumptions and models but generally offering more geographical precision, and these have been used in two recent studies. Thus, ground levels of radiation from $^{137}Cs$ in Sweden measured in May–October 1986 were used to divide the childhood population into 'unexposed' ($<10$ kBq $m^{-2}$) and 'exposed' ($\geqslant 10$ kBq $m^{-2}$) groups in order to compare leukaemia incidence before and after the Chernobyl accident (Hjalmars *et al*., 1994). The population weighted mean of environmental $^{137}Cs$ contamination in the exposed region (29 Bq $m^{-2}$) (which corresponds almost exactly to region 1 of Sweden in Figure 1) is the same as the UNSCEAR (1988) estimate (31 Bq $m^{-2}$) used as the basis of dose calculation in the present report. Six and a half years after the accident, 50 cases of leukaemia would be expected in the 'exposed' population in which individuals would have received a cumulative dose of around 1 mSv (Table I), giving an expected excess relative risk (ERR) of 3–5%, or 1–3 additional cases. It is clear that this Swedish study had no prospect of detecting a statistically significant excess incidence of childhood leukaemia unless the generally accepted assumptions about radiation leukaemogenesis are incorrect by a factor of 5–10. In a study using more appropriate epidemiological methods conducted in Finland (Auvinen *et al*., 1994), the average population dose (0.41 mSv over 2 years), derived from atmospheric sampling and whole-body counts, was partitioned in quintiles. The 95% confidence interval of the ERR estimate was $-0.27$ to $+0.41$. This range includes a leukaemogenic effect that is almost ten times greater than is conventionally assumed (0.045). The authors of these two studies imply that more accurate dose estimation confers an advantage over ECLIS. It seems obvious that any such advantage is heavily outweighed by lack of power to detect an effect. Ivanov *et al*. (1993) have compared incidence rates of childhood leukaemia in Belarus before (1979–85) and after (1986–91) the Chernobyl accident for regions with 'severe', 'intermediate' and 'least' radioactive contamination. No differences in incidence were reported. These results are difficult to evaluate, since no quantitative information on exposure is given.

A possible source of bias in this study is differential ascertainment of cases correlated with exposure; for example, as a result of improved detection of cases in heavily exposed populations living near Chernobyl. It is a potential problem for cancers that can exist in a 'latent' form and be diagnosed as a consequence of an active search for them. Ascertainment bias was suggested as a possible cause of the reported excess of thyroid cancer in children in Belarus (Beral and Reeves, 1992; Ron *et al*., 1992), although this now seems an unlikely explanation (Williams *et al*., 1993). There is no evidence of ascertainment bias in data for childhood leukaemia in Europe. Data submitted to ECLIS are checked for the traditional indicators of data quality used by cancer registries, including the proportion of leukaemia cases of unspecified cell type and the proportion with a histological diagnosis (Parkin *et al*., 1993). Although most registries do show small improvements in the latter indicator, there was, in fact, no association between the observed change (1987–91 *vs* 1980–85) and the estimated radiation dose.

The study will continue data collection for a period of 10 years post accident, so that the full potential of the excess radiation exposure can be studied. Future studies will examine in more detail the trends in incidence within different age groups and geographical regions, estimate the possible effects of migration and study separately urban and rural populations.

**Acknowledgements**
The authors acknowledge the contribution of Dr J Kaldor in establishing the ECLIS study. The study is supported by a grant from the Radiation Protection Programme, Directorate General for Science, Research and Development, Commission of the European Communities, Brussels (Contract FI3P-CT920062). The many sources of support for the cancer registries contributing to the ECLIS study are also gratefully acknowledged.

### References

AUVINEN A, HAKAMA M, ARVELA H, HAKULINEN T, RAHOLA T, SUOMELA M, SODERMAN B AND RYTOMAA T. (1994). Fallout from Chernobyl and incidence of childhood leukaemia in Finland, 1976–92. *Br. J. Med.*, **309**, 151–154.

BEIR V (COMMITTEE ON THE BIOLOGICAL EFFECTS OF IONIZING RADIATIONS). (1990). *Effects of Exposure to Low Levels of Ionizing Radiation*. National Academy Press: Washington, DC.

BERAL V AND REEVES G. (1992). Childhood thyroid cancer in Belarus (letter). *Nature*, **359**, 680–681.

DARBY SC, DOLL R, GILL SK AND SMITH PG. (1987). Long term mortality after a single treatment course with X-rays in patients treated for ankylosing spondylitis. *Br. J. Cancer*, **55**, 179–190.

DOLL R, BOICE JD, ESTÈVE J, SILINI G AND THIESSEN JW. (1990). Recommendations for research of an international panel of independent experts. In *Feasibility of Studies of Health Effects in Western Europe Due to the Reactor Accident at Chernobyl and Recommendations for Research* (Report EUR 12551 EN). Commission of the European Communities Directorate-General for Science, Research and Development: Luxembourg.

FRANCIS B, GREEN M AND PAYNE C (eds). (1993). *The GLIM System. Release 4 Manual*. Clarendon Press: Oxford.

HJALMARS U, KULLDORFF M AND GUSTAFSSON G. (on behalf of the Swedish Child Leukaemia Group). (1994). Risk of acute childhood leukaemia in Sweden after the Chernobyl reactor accident. *Br. Med. J.*, **309**, 154–157.

IVANOV EP, TOLOCHKO G, LAZAREV VS AND SHUVAEVA L. (1993). Child leukaemia after Chernobyl (letter). *Nature*, **365**, 702.

MCCULLAGH P AND NELDER JA. (1989). *Generalised Linear Models*, 2nd edn., Chapman & Hall: New York.

PARKIN DM (on behalf of the ECLIS Study Group). (1990). The European Childhood Leukaemia/Lymphoma Incidence Study. *Radiat. Res.*, **124**, 370–371.

PARKIN DM, CARDIS E, MASUYER E, FRIEDL HP, HANSLUWKA H, BOBEV D, IVANOV E, SINNAEVE J, AUGUSTIN J, PLESKO I, STORM HH, RAHU M, KARJALAINEN S, BERNARD JL, CARLI PM, L'HUILLIÉR MC, LUTZ JM, SCHAFFER P, SCHRAUB S, MICHAELIS J, MOHNER M, STANECZEK W, VARGHA M, CROSIGNANI P, MAGNANI C, TERRACINI B, KRIAUCIUNAS R, COEBERGH JW, LANGMARK K, ZATONSKI W, MERABISHVILI V, POMPE-KIRN V, BARLOW L, RAYMOND L, BLACK R, STILLER CA AND BENNETT BG (1993). Childhood leukaemia following the Chernobyl accident: The European Childhood Leukaemia–Lymphoma Incidence Study (ECLIS). *Eur. J. Cancer*, **29A**, 87–95.

PRESTON DL, KUSUMI S, TOMONAGA M, IZUMI S, RON E, KURAMOTO A, KAMADA N, DOHY H, THOMPSON DE, MABUCHI K, SODA M, MATSUI T AND NONAKA H. (1994). Cancer incidence in atomic bomb survivors. Part III: Leukaemia, lymphoma and multiple myeloma, 1950–1987. *Radiat. Res.*, **137**, S68–S97.

RON E, LUBIN J AND SCHNEIDER AB. (1992). Thyroid cancer incidence (letter). *Nature*, **360**, 113.

SHIMIZU Y, KATO H AND SCHULL WJ. (1990). Studies of the mortality of A-bomb survivors. *Radiat. Res.*, **121**, 120–141.

UNITED NATIONS SCIENTIFIC COMMITTEE ON THE EFFECTS OF ATOMIC RADIATION. (1988). *Sources, Effects and Risks of Ionizing Radiation, 1988 Report to the General Assembly, Annex D, Exposures from the Chernobyl Accident*. United Nations: New York.

UNITED NATIONS SCIENTIFIC COMMITTEE ON THE EFFECTS OF ATOMIC RADIATION. (1994). *Sources and Effects of Ionising Radiation UNSCEAR 1994, Report to the General Assembly with Scientific Annexes*. United Nations: New York.

WILLIAMS D, PINCHERA A, KARASGLOW A AND CHADWICK KH (eds). (1993). *Thyroid Cancer in Children Living Near Chernobyl* (Report EUR 15248 EN). Commission of the European Communities Radiation Protection Research and Training Programme: Luxembourg.